# On the role of bacterial metalloproteases in COVID-19 associated cytokine storm

**DOI:** 10.1186/s12964-020-00699-3

**Published:** 2021-01-13

**Authors:** László Földvári-Nagy, Tamás Schnabel, Gabriella Dörnyei, Tamás Korcsmáros, Katalin Lenti

**Affiliations:** 1 Department of Morphology and Physiology, Faculty of Health Sciences, Semmelweis University, 17. Vas str., Budapest, 1088 Hungary; 2 I. Department of Internal Medicine and Gastroenterology, Department of Orthopaedics - COVID Quarantine, ÉKC New Saint John’s Hospital, 1-3. Diós árok, Budapest, 1125 Hungary; 3 Earlham Institute, Norwich Research Park, Norwich, NR4 7UZ UK; 4 Quadram Institute Bioscience, Norwich Research Park, Norwich, NR4 7UQ UK

**Keywords:** IL-6, Cytokine storm, Metalloprotease, Bacteria, COVID-19

## Abstract

The cytokine release syndrome or cytokine storm, which is the hyper-induction of inflammatory responses has a central role in the mortality rate of COVID-19 and some other viral infections. Interleukin-6 (IL-6) is a key player in the development of cytokine storms. Shedding of interleukin-6 receptor (IL-6Rα) results in the accumulation of soluble interleukin-6 receptors (sIL-6R). Only relatively few cells express membrane-bound IL-6Rα. However, sIL-6R can act on potentially all cells and organs through the ubiquitously expressed gp130, the coreceptor of IL-6Rα. Through this, so-called trans-signaling, IL-6–sIL-6R is a powerful factor in the development of cytokine storms and multiorgan involvement. Some bacteria (e.g., *Serratia marcescens*,* Staphylococcus aureus*,* Pseudomonas aeruginosa*,* Listeria monocytogenes*), commonly considered to cause co-infections during viral pneumonia, can directly induce the shedding of membrane receptors, including IL-6Rα, or enhance endogenous shedding mechanisms causing the increase of sIL-6R level. Here we hypothesise that bacteria promoting shedding and increase the sIL-6R level can be an important contributing factor for the development of cytokine storms. Therefore, inhibition of IL-6Rα shedding by drastically reducing the number of relevant bacteria may be a critical element in reducing the chance of a cytokine storm. Validation of this hypothesis can support the consideration of the prophylactic use of antibiotics more widely and at an earlier stage of infection to decrease the mortality rate of COVID-19.

**Video abstract**

**Video abstract**

## Background

As of 16 October 2020 the worldwide mortality rate of COVID-19 caused by the SARS-CoV-2 virus is approximately 2.5% (49,727,316 confirmed cases, 1,248,373 deaths), but in specific countries, the current mortality rate can be much higher, e.g., Italy 4.5%, UK 4.2% [1]. The possibility that cytokine release syndrome or cytokine storm stands behind the severe cases of COVID-19 has been raised by several research groups [2–6]. Cytokine storm is a hyperreaction of the immune system, driven by a sudden increase in interleukin levels [7] often due to a sudden increase in viral load [8]. The cytokine storm has also been described previously for several infections e.g., H1N1 [9–11], H5N1 [12] influenza, MERS-CoV [13] and SARS-CoV [14] and we recently analysed the key cytokines involved in these infections [15].

One of the central protein molecules in the cytokine storm is interleukin-6 (IL-6). Monocytes, endothelial cells, fibroblasts, and activated Th2 cells produce IL-6 [16]. Viral infection induces IL-6 production through TNF-α [17–19].

**Fig. 1:**
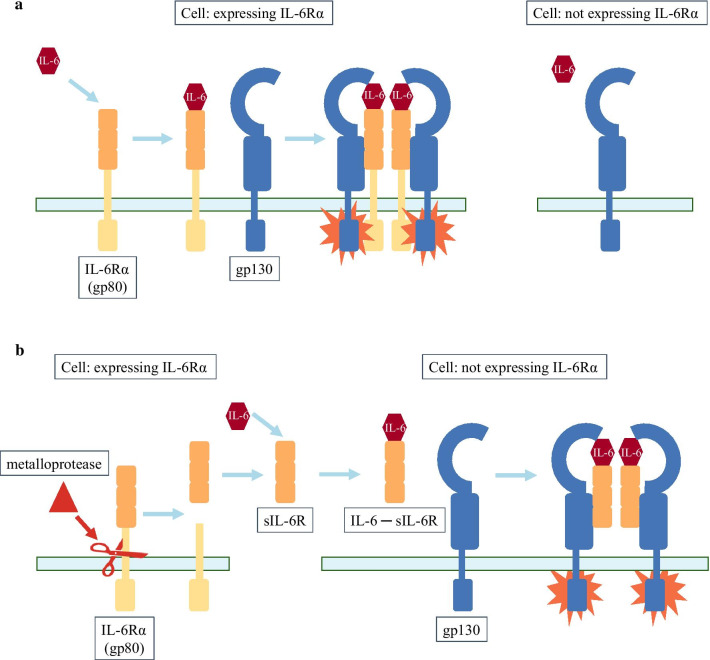
The mode of action of IL-6. **a** IL-6 binds to membrane-bound IL-6Rα (gp80). The IL-6–IL-6Rα complex binds to gp130 membrane receptor, which is responsible for signaling. IL-6 acts on cells expressing IL-6Rα. **b** Metalloproteases are able to cleave the exodomain of membrane-bound IL-6Rα (gp80). Bacterial exogenous metalloproteases are directly capable of IL-6Rα shedding. In addition, bacteria are able to solubilize IL-6Rα by activating endogenous metalloproteases. The resulting soluble sIL-6R binds IL-6 with the same affinity as membrane-bound IL-6Rα. The IL-6–sIL-6R complex is also able to bind to the gp130 membrane receptor on cells that do not express IL-6Rα, thus affecting organs that would not be affected by IL-6 and the cytokine storm. This mechanism may contribute to the development of multiorgan involvement in the cytokine storm

IL-6 acts on the IL-6 receptor (IL-6R). IL-6R is a protein expressed primarily in hepatocytes, megakaryocytes, and leukocytes [20, 21]. The IL-6R cell surface receptor complex consists of an 80 kDa IL-6 binding subunit, called gp80 (IL-6Rα), and a 130 kDa signaling subunit, called gp130 [22, 23]. The extracellular part is responsible for IL-6 binding. The resulting IL-6–IL-6Rα complex binds to gp130, causing gp130 to homodimerise (Fig. 1a). The intracellular region of homodimerised gp130 activates signalling [24, 25]. On the cells expressing IL-6Rα, IL-6Rα binds to gp130 membrane receptors, resulting in a cell-dependent response of IL-6 through activation of gp130 [21]. Gp130 is found not only on the surfaces of cells expressing IL-6Rα, but on nearly all cells in almost every organs [16, 26]. Thus, gp130 is also found on the surface of cells that, in the absence of the IL-6Rα receptor, are normally unaffected by IL-6.

The extracellular part of IL-6Rα can be cleaved by metalloproteases in a process called shedding [27, 28]. These metalloproteases can be endogenous metalloproteases such as ADAM10 and ADAM17 [29, 30] and exogenous bacterial metalloproteases [31] that cleave IL-6Rα to generate soluble IL-6Rα (sIL-6R). SIL-6R can bind IL-6 similarly as IL-6Rα, and promotes the IL-6 signal to gp130 (Fig. 1b) [32, 33]. Signaling via sIL-6R is called trans-signaling [34, 35].

Many pathogenic bacteria affect IL-6 signaling through several direct and indirect mechanisms. First, they can induce higher *IL-6* expression [36]. Second, they produce exogenous metalloproteases that stimulate the formation of sIL-6R by the shedding. Exogenous metalloproteases from many bacteria, such as *Bacillus subtilis*, *Serratia marcescens*, *Staphylococcus aureus*, *Pseudomonas aeruginosa*, *Listeria monocytogenes* are able to cleave IL-6Rα and other cell surface receptors [31, 37]. Bacteria-cleaved sIL-6R exhibits the same biological activity as those that are cleaved by endogenous metalloproteases or the membrane-bound IL-6Rα itself [31, 38]. The same activity includes that these sIL-6Rs are capable to induce IL-6 signaling in other, often distant cells through trans-signaling (Fig. 1b) [31, 33]. Third, bacteria can also be stimulators of endogenous receptor shedding by producing substances and toxins [38–40]. Bacterial toxins at very low, even at ng/ml concentrations, can induce a drastic (up to 50%) cleavage of IL-6Rα in a very short time (in 10 min) resulting in measurable physiological effect [38]. For example, the toxins streptolysin O by Group A Streptococcus (GAS) and hemolysin by *Escherichia coli* can induce the release of IL-6Rα from human monocytes and macrophages [38, 41]. The lipoteichoic acid toxin of *S. aureus* stimulates, for example, ADAM10, which not only solubilizes IL-6Rα, but also influences EGF receptor activation by playing a role in HB-EGF ectodomain shedding. Through the HB-EGF ectodomain shedding, the toxin of *S. aureus* activates the production of mucin in the lung, leading to airway obstruction, which is also a problem in COVID-19 infection [42]. *S. aureus* and *P. aeruginosa* induce IL-6Rα shedding by stimulating ADAM17 [36]. Because of all the reasons above, it is highly important to acknowledge that in a number of bacterial infections the exogenous or endogenous metalloprotease levels and their activity are elevated [31, 38]. Even if the bacteria do not manifest a disease, it can cause high levels of sIL-6R, and through the formation of IL-6–sIL-6R complexes, it can significantly increase the number of cells and organs responding to IL-6 signaling (Fig. 1b) [43–45].

## Presentation of the hypothesis

Based on the known direct and indirect effect of certain bacterial species to increase the level of sIL-6R [31, 38], and the previously published role of the IL-6‒sIL-6 complex in forming cytokine storms [46], here we propose that these bacterial species could be key contributors to induce the IL-6 mediated cytokine storms.

Many bacterial species with exogenous metalloproteases that increase the level of sIL-6R are also the most common co-infections in viral pneumonia [47]: *S. aureus*, *Klebsiella pneumoniae*, *P. aeruginosa*, and *S. marcescens*. We hypothesise that the presence of any of these or similar bacteria at the time of a viral infection can lead to an increased level of sIL-6R and exacerbation of the severe disease processes, including multiorgan involvement and cytokine storms. Thus, the presence of these bacteria should be considered not only as cause of co-infections but as predisposing factors, which may worsen the outcome of SARS-CoV-2 virus infection by enhancing IL-6 mediated signaling.

Our hypothesis fits perfectly with the two-hit model of systemic inflammation caused by lung injury leading to cytokine storm. Recently, the two-hit model was applied to SARS-CoV-2 infection, where following lung injuries, as a first hit cytokine (e.g., IL-6) release is increased, then the released cytokines and other factors induce inflammation in the lungs [48]. Through the stimulation of bone marrow, cytokines induce further lung inflammation. This feedback loop, as a second hit results in a cytokine storm [48]. We point out here that the initial lung injury can be the result of or associated with the preliminary presence in the host of bacterial species capable of inducing metalloproteases.

## Testing the hypothesis

The proposed hypothesis is complex to test but it is possible. One approach should aim to demonstrate the role of a bacterial predisposition, in particular the role of metalloproteases in increasing the cytokine storm effect upon SARS-CoV-2 infection through increased IL-6/sIL-6R levels. Ideally, this experiment should be in vivo to demonstrate the physiological relevance of the proposed model. Another approach should validate the relevance of these findings in infected human lung models, which recapitulate COVID-19.

To demonstrate the physiological relevance of our hypothesis, rodent models would be adequate, such as hACE2 transgenic mice or hamsters, as they generate similar immune responses to humans upon SARS-CoV-2 infection [49]. In this in vivo experiment, animals would be pre-treated with one of the bacterial species listed above (e.g., *S. aureus*, *P. aeruginosa*, *S. marcescens*), followed by SARS-CoV-2 infection. The experiment would contain the following conditions: (1) normal control (no treatment), (2) only bacterial pre-treatment, (3) only SARS-CoV-2 infection, (4) both bacterial pre-treatment and SARS-CoV-2 infection. Furthermore, we propose to generate a bacterial strain that lacks its known and key metalloprotease (e.g., *S. marcescens* metalloproteinase) and use it in additional conditions to demonstrate the metalloprotease dependency of the observed changes. We suggest measuring the serum level of sIl-6r and cytokine storm markers (Il-8, Il-18, Ang-2 and von Willebrand factor) [50] in five time points over 3 weeks to capture the dynamics of the cytokine storm. In addition, while it is not essential to directly prove the hypothesis, after sacrificing the animals, key organs such as lung, kidney, brain or the intestine can be used to gain deeper understanding of local tissue damage in the various conditions. The measurements from each condition can be compared to establish: (a) the effect of bacterial pre-treatment on the dynamics of cytokine storm development (using condition 3 vs 4, and condition 2 as a control); (b) the role of a bacterial metalloprotease in the cytokine storm dynamics (comparing measures upon pre-treatment with wild-type and metalloprotease lacking bacterial strains in condition 2 and condition 4 separately. We assume to prove our hypothesis, comparison of condition 3 versus 4 will result in decreased time and potentially increased level for cytokine storm markers, and these levels would correlate with the serum level of sIl-6r.

To validate the relevance of the hypothesis for human COVID-19 and to confirm the specific role of the metalloproteases in this process, we propose to use human lung organ chip systems, such as the one created recently by Zhang et al. [51]. Here using similar conditions as proposed above, the production of Ang-2 and von Willebrand factor from the endothelial cells can be measured and compared between the same conditions. Furthermore, this experimental model will enable us to add only specific metalloproteases or bacterial toxins to the system, like SMP (*Serratia marescens* metalloproteinase), hemolysin (*E. coli*) or Streptolysin O (Group A Streptococcus) and not living bacteria. With this approach we can eliminate the co-infection related complex effect proposed in the previous experiment, and address the role of these specific metalloproteases (one in each experiment with three conditions based on previous publications [38]. Finally, to validate the proposed mechanism itself, this experimental system also allows to model the effect of increased IL-6 even without SARS-CoV-2 infection. By adding IL-6 to the microfluidic system with and without the presence of bacterial pre-treatment, one can measure sIL-6R levels and key IL-6 target gene expression changes with qPCR.

## Implications of the hypothesis

Validation of our hypothesis will support efforts to reduce the risk of developing life-threatening conditions, for example by initiating appropriate antibiotic therapy prophylactically in the early stages of infection. For those at risk and/or those with underlying health conditions it would be worthwhile to reconsider the treatment recommendations to minimize sIL-6R shedding much earlier, in the initial stage of the disease. We recommend considering the administration of antibiotics to reduce the likelihood of developing a cytokine storm.

Several studies suggest that treatment of COVID-19 patients with antibiotics may be important for the prevention and treatment of bacterial co-infections [52–54]. However, treatment protocols/recommendations recommend antibiotic treatment in case of bacterial co-infections [47, 55, 56], often in patients with developing severe conditions or in the case of multiorgan failure.

Medical recommendations are usually very cautious about the use of antibiotics due to the risk of developing antibiotic-resistant bacterial strains. In addition, caution is advised as some antibiotics act by stimulating the immune response (increasing the amount of IL-6, IL-1, TNF-α), which can potentially induce a cytokine storm, thus helping to develop a life-threatening condition [57].

Based on the physiological processes mentioned above, it is probable that an earlier introduced antibiotic treatment to a wider population may contribute to a better prognosis. This may be particularly important in virus infected closed communities (e.g., nursing homes, non-COVID-19 hospital wards).

In case of clinical approval of the efficacy of our suggestion, it would be important to test similar antibiotic treatment for other viral diseases threatening to develop a cytokine storm (such as H1N1, MERS-CoV, SARS-CoV), and after confirmation include the antibiotics in the respective treatment protocol.

## Limitations and further considerations

Further analysis on the effectiveness of the empirical antibiotic treatment is recommended in clinical studies. Considering the huge variety of chronic diseases and the various use of antibiotic regiments, it is going to be difficult to randomise the required data in an extensive clinical study. Therefore, we suggest data examination through the means of meta-analysis and/or systematic review. Planning of such study would require sufficient general data of the patients, their chronic diseases and the antibiotics used prior to and during their treatment. There are multiple integrated global resources already available for researcher, like the commercial IBM MarketScan® [58], Clinical Practice Research Datalink [59] and Premier® Healthcare Database (PHD) [60]. They contain data of patients currently under antibiotics for a different reason and their hospitalization rates, CRP levels, ventilation and need of ICU. In the near future, analysing these databases could indicate the possible role of bacterial infection in the severity of COVID-19. While these meta-analysis studies would provide indirect evidence, they could be complemented with those direct validatory experiments we propose above. Data extraction and randomisation from retrospective and case studies would not require any additional clinical testing.

The higher level of sIL-6R proved to be correlated with diseases e.g., asthma [61] and dermatitis [62]. It is possible to measure the sIL-6R level from serum or sputum [63] of patients at a very early stage of infection or those at risk of SARS-CoV-2 infection. These data may provide information to define possible predictive sIL-6R risk levels. Defining key sIL-6R levels that can predict the potential outcome of the infection would help to plan the most effective, personal treatment for each patient. However, it is important to note that the sIL-6R level can be influenced by many circumstances (advanced stage of COVID-19 infection, other diseases, genetic differences, alternative splicing etc.). Therefore, the measurement of sIL-6R level can be a useful and effective predictive tool but not specific to measure the influence of bacterial induced metalloproteases.

In several countries a higher rate of mortality associated to COVID-19 is observed in Black, Asian and Minority Ethnic (BAME) communities [64, 65]. There is no explanation for the difference yet. Among the possible reasons, a relevant difference in the bacterial flora cannot be ruled out. However, the polymorphism of the IL-6 and IL-6R genes in different ethnic groups is more likely to be behind the observed difference [66, 67]. These polymorphisms can be associated with differences in the incidence of certain diseases (e.g., atrial fibrillation, cancer) between ethnic groups [67–69]. Our hypothesis study does not cover the study of such differences, only the description of the hypothesised mechanism and the prevention or alleviation of diseases associated with cytokine storms, respectively. However, the problem of different mortality of ethnic groups is important from a clinical point of view, so it would be worthwhile to examine this in more detailed, subsequent studies.

As it was shown for COVID-19 infection that levels of IL-6, along with IL-8 and TNF-α, were powerful predictors of severity and survival at the time of hospitalization [70], using IL-6 inhibitors could be a promising therapy against sever COVID-19. At present however it is too early to declare whether IL-6 inhibitors, such as Tocilizumab (TCZ) will prove to be efficient. In a number of single centre studies TCZ seems to be effective [71–73] but other studies do not seem to report positive results [74, 75]. The systematic reviews and meta-analysis data still have a high grade of bias due to the high variation of therapeutic schemes [76]. If any gp130 antagonist or any IL-6 inhibitor/antibody treatment would prove to be efficient in the future, it would support our theory.

Patients may face serious conditions and complications that are typically associated with COVID-19 infection at a later stage (e.g., COVID-19 Associated Lung Injury, Diffuse Alveolar Damage, edema, respiratory failure etc.) [48]. These diseases can lead to serious, life-threatening conditions and can induce a cytokine storm as a consequence of lung damages and inflammation. In this paper, we wanted to draw attention to the risk factors in the very early stages of COVID-19 infection and potential options for their elimination. While there are multiple reasons that can lead to the development of a cytokine storm especially in the later stages of COVID-19 infection, bacterial induced elevation of sIL-6Rs as a result of early and preceding bacterial infections could be one of the causes that can be well controlled. Removing these bacterial infections will not eliminate all the causes, especially those caused by subsequent severe conditions, but it can have an important preventive effect in a hopefully significant part of the cases or groups at risk.

## Data Availability

Not applicable.

## Abbreviations

ADAM10: A Disintegrin and metalloproteinase domain-containing protein 10
ADAM17: A Disintegrin and metalloproteinase domain-containing protein 17
Ang-2: Angiopoietin-2
BAME: Black, Asian and Minority Ethnic
COVID-19: Coronavirus disease
EGF: Epidermal growth factor
GAS: Group A Streptococcus
gp80: (=IL-6Rα) IL-6 binding subunit of interleukin-6 receptor
gp130: Signaling subunit of interleukin-6 receptor
H1N1: Hemagglutinin Type 1 and Neuraminidase Type 1 influenza strain
H5N1: Hemagglutinin Type 5 and Neuraminidase Type 1 influenza strain
hACE2: Human Angiotensin-converting enzyme 2
HB-EGH: Heparin-binding EGF-like growth factor
IL-1: Interleukin-1
IL-6: Interleukin-6
IL-6R: Interleukin-6 receptor
IL-6Rα: IL-6 binding subunit of interleukin-6 receptor
Il-8: Interleukin-8 (mouse)
Il-18: Interleukin-18 (mouse)
MERS-CoV: Middle East Respiratory Syndrome Coronavirus
qPCR: Quantitative Polymerase Chain Reaction
SARS-CoV-2: Severe acute respiratory syndrome coronavirus 2
sIL-6R: Soluble interleukin-6 receptor
SMP: *Serratia marescens* Metalloproteinase
TCZ: Tocilizumab
Th2: T helper cell 2
TNF-α: Tumour Necrosis Factor α
